# Genotoxic evaluation of the River Paranaíba hydrographic basin in Monte Carmelo, MG, Brazil, by the *Tradescantia* micronucleus

**DOI:** 10.1590/S1415-475738420150117

**Published:** 2015

**Authors:** Carlos F. Campos, Boscolli B. Pereira, Edimar O. de Campos-Junior, Eduardo F. Sousa, Henrique N. Souto, Sandra Morelli

**Affiliations:** 1 Departamento de Biologia Celular, Fundação Carmelitana Mário Palmério, Monte Carmelo, MG, Brazil; 2 Instituto de Geografia, Universidade Federal de Uberlândia, Uberlândia, MG, Brazil; 3 Instituto de Genética e Bioquímica, Universidade Federal de Uberlândia, Uberlândia, MG, Brazil

**Keywords:** biomonitoring, genotoxicity, micronucleus, Tradescantia pallida

## Abstract

Pollutants have adverse effects on human health and on other organisms that inhabit or use water resources. The aim of the present study was to assess the environmental quality of three watercourses in Monte Carmelo, MG, Brazil, using the micronucleus test on *Tradescantia*. For each treatment, 15 plants were exposed to water samples for 24 h. The control group was exposed to formaldehyde (0.2%) and the negative control to Hoagland solution. Subsequently the plants were placed in Hoagland solution for 24 h to recover. Cells were stained with 2% acetic carmine and examined by light microscopy. Three hundred tetrads were analyzed per slide. The frequency of genotoxic alterations was expressed as the number of micronuclei per 100 tetrads, and the groups were compared by ANOVA. At all sample sites for each watercourse significant genotoxicity indices were observed. The results suggest that in the Mumbuca creek, the current situation of effluent discharge should be reconsidered by the municipal environmental authorities. The increase in micronucleus frequency denoted for water samples of the Mumbuca creek, Lambari river and Perdizes river emphasizes the need to adopt environmental vigilance strategies, such as biological monitoring.

## Introduction

The World Health Organization (WHO) states that water is essential to sustain life, and is a substance that should necessarily be available to all in adequate, safe and accessible form. Improving access to potable water of appropriate quality is fundamental to health throughout lifetime. Thus microbiological antisepsis, chemicals, radiological compounds, flavor, odor, appearance parameters and catchment systems, treatment and distribution should be monitored periodically to guarantee an adequate water quality control (WHO, 2011).

Resolution N° 357 of 2005, of the Brazilian National Environment Council (CONAMA) establishes water quality standards in virtue of substance concentrations that, when surpassed, can affect the population)s health, safety and well-being, besides causing damage to fauna and flora and the environment in general (CONAMA, 2005).

Aquatic environments have been highly polluted in function of effluent discharge of diverse origins. Treated or not, these can be domestic, mining and industrial effluents or water run-off in agricultural areas (Köck-Schulmeyer *et al.*, 2013) that commonly contain contaminating agents such as heavy metals, aromatic polycyclic hydrocarbons and various agricultural chemicals (Demarini *et al.*, 1996). These pollutants have adverse effects on human health and on other organisms that inhabit or use water resources (Isidori *et al.*, 2003). Thus, using biomonitoring programs for environmental quality control is necessary because it contributes important information on potential risks to the environment and biota.

Magalhães and Ferrão-Filho (2008) suggested that the individual response of an organism is a good indication of environmental disturbances, because responses to stressful agents and contaminants occur at biochemical, cellular and physiological levels in an integrated manner. They can be analyzed before these effects become manifest in populations, communities and ecosystems, so they are a good alternative to prevent/anticipate more serious damage to the environment (Bortolotto *et al.*, 2009).

In this sense, a widely used approach is to measure environmental disturbances by means of physiochemical analyses that provide data about pollution levels. However, such analyses are strictly quantitative in terms of compound levels, but are not sufficient to indicate possible risks for the biota. Hence, being a methodology that lacks complements to guarantee efficiency in environmental quality analysis (Dalzell *et al.*, 2001), it is necessary to also analyze and quantify polluting activities and sources possibly related to genotoxic effects (Grant, 1982).

A viable alternative is to use plant biomonitoring to assess physicochemical factors and their correlations with chromosomal alterations/aberration frequencies, in addition to mitotic events (Monarca *et al.*, 1999). The plant genus *Tradescantia* has become established as a widely used system to show genetic damage and is considered a model in this context (Misík *et al.*, 2007).

Genetic alterations that occur in *Tradescantia* can be detected by analyzing chromosome aberrations/breaks in germinated cells. These can be micronuclei (MN) that are portions of chromatin close to the nucleus, derived from acentric chromosomes, induced by clastogenic (chromosome breaks) and aneugenic effects that induce aneuploidy or abnormal segregation (Almeida Neto *et al.*, 2005; Mielli *et al.*, 2009), caused by agents that may be present in different environments such as water, air and soil (Gopalan, 1999; Majer *et al.*, 2005).

The *Tradescantia* micronucleus test (TRAD-MCN) is one of the most used assays to reveal genotoxic and/or carcinogenic effects in higher organisms. The basis of this test is the formation of micronuclei derived from chromosome breaks that occurred during meiosis, *i.e.* the pollen grain formation phase of *Tradescantia* spp*.* flowers (Pereira *et al.*, 2013).

For more than three decades, data and results obtained from experiments with *Tradescantia* have been comparable, clear and reliable. The TRAD-MCN has many advantages, because it is a low-cost and very versatile method that makes it suitable for application in developing countries (Grant, 1994). As a biological monitoring method it is employed to complete the results of physicochemical monitoring, because it reveals *in vivo* effects caused by pollutants present in the environment*.* The aim of the present study was to use the micronucleus test with *Tradescantia pallida* (Rose) D.R. Hunt *var. purpurea* to biomonitor the water quality at eight sites along three water courses (Mumbuca creek and the Lambari and Perdizes rivers) to reveal human actions in the town of Monte Carmelo, Minas Gerais, Brazil.

## Material and Methods

### Sampling sites

The town of Monte Carmelo, MG, Brazil, is in the region of the Upper Paranaíba hydrographic basin (South 18°44′5″; West 47°29′47″) of the State of Minas Gerais, Brazil. The last demographic census reported that it has 45.772 inhabitants and a total area of 1.343.035 km^2^ (IBGE, 2010). It is in a predominantly Cerrado biome with some areas of Atlantic Rainforest. The climate is of the Aw and Cwa type in the Köppen classification, with warm, wet summers from November to April and a cool dry winter from May to October (EMBRAPA, 2004). The main uses of the water resources in this town are: irrigation, animal drinking, public supply and human consumption, and industrial use (IGAM, 2009).

**Figure 1:**
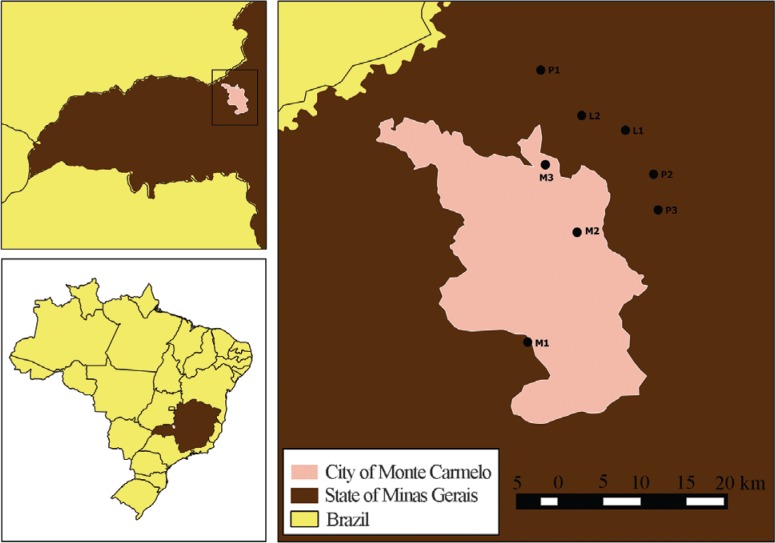
Water collection sites located in the town of Monte Carmelo, Minas Gerais, Brazil. Mumbuca creeck (sites M1, M2 and M3), Lambari river (sites L1 and L2) and Perdizes river (sites P1, P2 and P3).

Water was collected following the model proposed by the São Paulo State Environmental Company (CETESB, 2011). Water was collected at the beginning of July 2014 on a single day. The points monitored (Figure 1) for each water resource were:

Mumbuca creek - *M1:* close to the headwaters (geographical coordinates: 18°44′19.60″ S and 47°29′47.40″ W); *M2:* urban environment (geographical coordinates: 18°43′25.10″ S and 47°29′40.10″ W); *M3:* urban environment (geographical coordinates: 18°42′31.90″ S and 47°29′37.80″ W).

Lambari river - *L1:* close to the headwaters in a rural environment (geographical coordinates: 18°43′19.60″ S and 47°28′29.10″ W); *L2:* rural environment (geographical coordinates: 18°42′10.30″ S and 47°28′27.70″ W).

Perdizes river - *P1:* rural environment (geographical coordinates: 18°39′48.70″ S and 47°29′14.30″ W); *P2:* highway perimeter in front of the municipal landfill (geographical coordinates: 18°40′30.40″ S and 47°28′7.10″ W); *P3:* rural environment behind the municipal landfill (geographical coordinates: 18°41′48.10″ S and 7°26′57.30″ W).

### Water quality index (WQI)

The water courses in the Paranaíba river basin are classified as class 2 according to CONAMA Resolution number 357 of 2005 for freshwater classification. Data from other analytical segments (physicochemical parameters determination) were obtained from data supplied by the Minas Gerais Water Management Institute (IGAM). According to the IGAM, the following concepts are defined according to the weighting of the parameters for water quality: Excellent (90 < IQA ≤ 100), Good (70 < IQA ≤ 90), Medium (50 < IQA ≤ 70), Poor (25 < IQA ≤ 50) and Very bad (0 < IQA ≤ 25). Concerning the data for the water quality index (WQI) obtained from the IGAM, a decrease in quality in the aquatic environments was inferred in the short term for the town under study. According to IGAM (2011, 2012) the surface quality of the water courses, generally, was considered Medium with little contamination by diverse toxic agents in 2011 and 2012, but was classified the next year for the same parameters as being of Poor surface quality and with medium contamination by diverse toxic agents.

### Preparation of *Tradescantia* and treatment procedure

*Tradescantia pallida* plants used in the present study were grown in a greenhouse at the Fundação Carmelitana Mário Palmério - FUCAMP, following a protocol proposed by Ma *et al.* (1994). The ambient temperature was kept between 16 °C at night and 26 °C during the day, and the relative humidity was controlled at around 60-80%. A 16 h daily photoperiod was applied to induce flowering. The plants were grown in 1 L pots adapted for the exposition method. Fertilization, irrigation and the spontaneous mutation rate for the plant stock were systematically controlled. Producing cuttings from the same plant guaranteed the isogenicity of the samples.

The bioassay was performed following the recommendations given by Ma (1983) with modifications. Formaldehyde at 0.2% was used as positive control, and treatments with Hoagland)s solution were used as negative control. Commercial plant substrates were used and vermiculite (Bioplant, Minas Gerais, Brazil).

According Ma *et al.* (1994), flowerbeds with at least 15 plants were used for the experiment per study site, including the positive and negative controls. Stems measuring 15 cm in length with young flowers were cut and placed in Hoagland)s solution (Hoagland and Arnon, 1950) for 24 h for acclimation, followed by treatment for 24 h in water samples collected at the eight sampling points, or the control solutions, respectively. Subsequently the plants were placed in Hoagland)s solution for 24 h to recover for the subsequent TRAD-MCN experiment.

After the acclimation, treatment and recovery steps, young flowers were taken from the stems and immediately fixed in Carnoy solution (3:1 ethanol and glacial acetic acid) for 24 h and then preserved in 70% ethanol until the time of analysis. Anthers collected from the buds were mashed with a glass stick on a microscope slide and a drop of acetic carmine was added for staining. They were mashed and cleaned (discarding the anther fragments) and the slide covered with a cover slip and quickly heated to 80 °C to fix the stain on the TETRADS. Micronucleus frequencies were calculated as the number of micronuclei per 100 tetrads analyzed. For each monitored site, 20 flower buds with pollen grain cells in the tetrads state were used. Five slides were produced for each site, and 300 tetrads per study site were assessed for the presence of micronuclei under a light microscope with 400 times magnification (Ma *et al.*, 1994).

### Statistical analysis

Analysis of variance (ANOVA) followed by a Tukey post hoc test was used to determine the significance of differences among the evaluated sampling points (Callegari-Jacques, 2006), with p ≤ 0.05 considered statistically significant.

## Results and Discussion

The micronucleus frequency at all water sample points was significantly higher when compared to the respective negative control (ANOVA, Tukey, p < 0.05). The data presented in Table 1 show increase in micronucleus frequency at all the points analyzed, reflecting an increase in the genotoxic potentiality along the copa water courses. Studies by Costa *et al.* (2014) emphasized the decrease in water quality in function of domestic and industrial effluent discharge along the course of a river, regardless of its relief.

**Table 1:** Micronucleus frequency at the sampling points in the Paranaíba river hydrographic basin, Monte Carmelo, MG, Brazil.

Collection sites	Frequency of MN/100 cells ± SD
Negative Control	2.4^a^ ± 1.14
L1	13.60^b^ ± 1.78
L2	14.2^b^ ± 2.96
M1	6.6^b^ ± 1.34
M2	8.8^b^ ± 2.49
M3	13.8^b^ ± 2.58
P1	8.2^b^ ± 1.74
P2	7.2^b^ ± 1.48
P3	10.6^b^ ± 1.81
Positive control	24.4^c^ ± 2.07

Different letters indicate statistically significant differences among MN frequencies (ANOVA, Tukey test, p < 0.05).

Table 2 shows increase in parameters over three years that negatively influenced the municipal WQI and discharge of sewage is postulated as a possible pollutant source (IGAM, 2013). The decrease in the quality in the aquatic environments, confirmed by the biological parameters used, can be further corroborated by other parameters (Table 2).

**Table 2:** Parameters above the legal limit, according to the Brazilian normative resolution (Minas Gerais, 2008) in percentage, in the Paranaíba river hydrographic basin in Monte Carmelo, MG, Brazil.

Parameters	Violation percentage (%)	Sampling		
		2011	2012	2013
Biochemical oxygen demand	72	2.00	3.10	8.60
*Escherichia coli*	15900	-	-	> 160000
Total iron	177	0.37	0.18	0.83
Total phosphorus	640	0.19	0.25	0.74
Manganese	13	0.05	0.06	0.11
Waterborne ammonia	85	1.53	2.08	6.86
Dissolved oxygen	85	6.70	4.80	2.70
Surface-active substances	106	< 0.10	0.39	1.03

Source: IGAM (2013).

Regarding the Perdizes river, a municipal landfill is located exactly between sites P2 (before treated leachate discharge) and P3 (after treated leachate discharge). Leachate formation is among the great problems inherent to landfills. Leachate is a dense, dark-colored liquid with an unpleasant odor that has varied physicochemical composition due to the state of decomposition of the residues and its original constituents (Tavares BFD, 2011, Thesis, Engenharia Ambiental na Escola Politécnica, Universidade Federal do Rio de Janeiro, Rio de Janeiro). After aerobic and anaerobic treatment, leachate is discharged directly into this watercourse, but even after treatment, some parameters are still above the tolerable limit (Table 3) and give a poor WQI for both treated leachate (WQI - 25.21) and sampling point P3 (WQI = 35.76). Table 3 shows six parameters obtained by physicochemical analyses. These parameters present values at site P3 above the desirable limit post-leachate and sulfide treatment, in accordance with a Brazilian normative resolution (CONAMA, 2005).

**Table 3:** Physiochemical parameters of site P2, treated leachate, and site P3.

Parameter (unit)	P2	Leachate	P3
pH (UpH)	6.7	9.2*	5.4
Dissolved oxygen (mg/L)	7.35	5.15*	7.43
Biochemical oxygen demand (mg/L)	25.33	1.375.49*	108
Sulfide (mg/L)	0.11	1.22*	1.05*
Cadmium (mg/L)	< 0.2	0.23*	< 0.2
Coliforms (NMP/100 mL)	700	2476*	576

* Substances above the desirable limit (CONAMA resolution Nr. 357 of 2005).

The genotoxicity of the tested samples, especially in the Mumbuca creek, can be attributed to the management of effluents from the town, which directs all the residues to the sewage treatment station that was recently built. Currently, this facility does not have the capacity to meet all the needs of the town, which leads to irregular discharge of effluents into the watercourse (DMAE, 2014). Thus, as was shown by the TRAD-MCN test, effluents of various origins (domestic, industrial and pluvial) can mix and become complex, causing genotoxic potential for the water samples taken at the study sites.

As mentioned previously, irrigation is among the main activities using water resources in the municipality. Livestock rearing and agriculture are the main economic activities in the municipality. According to IBGE (2012a,b), coffee production (*Coffea arabica* L.) was over 29 tons in 2012, and there are about 56,000 heads of cattle in the area. In this sense, over time, the fragmentation of local ecosystems becomes inevitable, especially by coffee monocultures situated in regions close to watercourses. The Lambari and Perdizes rivers are close to the areas planted mainly with coffee, among other crops, along their courses, including in the area of the sample points of the present study. As pointed out by Köck-Schulmeyer *et al.* (2013), water run-off that contains agricultural chemicals may damage the nearby watercourses. The use of agricultural chemicals is routine in coffee plantations, therefore, as indicated by Sanches *et al.* (2003), the use of pesticides implies environmental contamination, especially of surface water bodies and underground water sheets, as well as an increase in fish and bird mortality and food contamination. Genotoxicity at these points of the watercourse can be attributed for the most part to the use of agricultural chemicals and the inappropriate effluent discharge from rural properties.

For the Perdizes river there is a prevalence of undesirable values for sulfides at site P3, after the discharge point where the treated leachate enters the river body and is diluted. This is possibly a reflection of the methodology used in the treatment, because by anaerobic treatment, metals may precipitate in the form of metallic sulfides. The biological treatment used generally leads to improvement in the parameters for untreated leachate, but the high concentration of some metals, especially heavy metals, can lead to biological inhibition, so that chemical precipitation should be associated with the biological treatment (Iwai CK, 2005, Master)s thesis, Faculdade de Engenharia, Universidade Estadual Paulista, Campus Bauru).

The WQI data, specifically those for treated leachate and at site P3, suggest that the biological treatment used should undergo reconsideration, as alone it does not present enough improvement in the leachate parameters, as required by the environmental agencies. Crude leachate samples were not assessed because they have recognized genotoxic properties (Obidoska and Jasinska, 2008). The treated leachate value was WQI = 25.21, which represents a value between Poor and Very bad on the WQI classification scale by IGAM, reaffirming the need to associate other methodologies for leachate treatment, as proposed by Nunes *et al.* (2011).

The first collection site on the Perdizes river (P1), did not differ statistically from P3. In this case, although P1 was distant and possibly did not suffer directly from the influence of the municipal landfill, the possibility for similar genotoxicity at both the sites can be explained generically by the proximity of this sampling site to the cropped regions, as occurs also on the Lambari river, indicating recurrent use of agricultural chemicals. As reported by Oliveira (Oliveira DA, 2010, Thesis, Instituto de Geografia, Universidade Federal de Uberlândia), the vegetation on the Perdizes river banks is little preserved and the use of agricultural chemicals is common in the surroundings of this watercourse.

In conclusion, our results suggest that in the Mumbuca creek the current situation of effluent discharge should be reconsidered by the municipal environmental authorities, seeking the improvement of the system. This should consider the variability and complexity of the existing effluents (rainfall, domestic and industrial) and the influence they have on the conservation and environmental quality of the watercourse. On the Lambari river, the observed increase in micronucleus frequency at L1 and L2 emphasizes the need to adopt environmental vigilance strategies, such as biological monitoring to assess the direct influence of agricultural chemicals at these and other sites on the stream, and to direct the effluents from rural properties in this region to the municipal treatment station. For the Perdizes river the inference is that leachate discharge negatively influences the environmental quality of the River Perdizes, especially so at the site P3.

## References

[B1] Almeida JX, Medeiros FPM, Melo AJM, Silva JC, Dantas JP (2005). Avaliação do efeito mutagênico da palma forrageira (*Opuntia
fícus-indica* Mill) através do teste de micronúcleos em medula óssea de
ratos (*Rattus novergicus*, linhagem Wistar) *in
vivo*. Rev Biol Ciênc Terra.

[B2] Bortolotto JBT, Bertoldo FZD, Silveira TM, Silvano DJ, Pitch CT (2009). Evaluation of the toxic and genotoxic potential of landfill leachates
using bioassays. Environ Toxicol Pharmacol.

[B3] Callegari-Jacques SM (2006). Bioestatística: Princípios e Aplicações.

[B4] Costa GM, Cassanego MBB, Petry CT, Benvenuti T, Kieling-Rubio MA, Rodrigues MAS, Droste A (2014). Monitoramento químico e do potencial genotóxico para o diagnóstico da
qualidade de corpos hídricos. Rev Bras Ciênc Ambient.

[B5] Dalzell DJ, Alte S, Aspichueta E, De La Sota A, Etxebarria J, Gutierrez M, Hoffmann CC, Sales D, Obst U, Christofi N (2001). A comparison of five rapid direct toxicity assessment methods to
determine toxicity of pollutants to activated sludge. Chemosphere.

[B6] Demarini DM, Shelton ML, Bell DA (1996). Mutation spectra of chemical fractions of a complex mixture: Role of
nitroarenes in the mutagenic specificity of municipal waste incinerator
emissions. Mutat Res.

[B7] EMBRAPA (2004). Boletim de Pesquisa e Desenvolvimento: Levantamento de
Reconhecimento de Média Intensidade dos Solos da Região do Alto Paranaíba, Minas
Gerais.

[B8] Gopalan HNB (1999). Ecosystem health and human well being: The mission of the
international programme on plant bioassays. Mutat Res.

[B9] Grant WF (1982). Chromosome aberration assays in *Allium*. A report of
the U.S. Environmental Protection Agency Gene-Tox Program. Mutat Res.

[B10] Grant WF (1994). The present status of higher plant bioassays for detection of
environmental mutagens. Mutat Res.

[B11] Hoagland DR, Arnon DI (1950). The water-culture method for growing plants without
soil.

[B12] IGAM (2009). Monitoramento da Qualidade das Águas Superficiais da Bacia do
Rio Paranaíba.

[B13] IGAM (2012). Monitoramento da Qualidade das Águas Superficiais no Estado de
Minas Gerais, Relatório trimestral, 3° trimestre de 2012.

[B14] Isidori M, Ferrara M, Lavorgna M, Nardelli A, Parrela A (2003). In situ monitoring of urban air in Southern Italy with the
*Tradescantia* micronucleus bioassay and semipermeable membrane
devices (SPMDs). Chemosphere.

[B15] Köck-Schulmeyer M, Villagrasa M, De Alda ML, Céspedes-Sánchez R, Ventura F, Barceló D (2013). Occurrence and behavior of pesticides in wastewater treatment plants
and their environmental impact. Sci Total Environ.

[B16] Ma TH, Kolber A, Wong T, Grant L, Dewoskin R, Hughes T (1983). *Tradescantia* micronuclei (Trad-MCN) test for environmental
clastogens. *In Vitro* Toxicity Testing of Environmental Agents, Part
A.

[B17] Ma TH, Cabrera GL, Chen R, Gill BS, Ssandhu SS, Vandenberg AL, Alamone MF (1994). *Tradescantia* micronucleus bioassay. Mutat Res.

[B18] Magalhães DP, Ferrão AS (2008). A ecotoxicologia como ferramenta no biomonitoramento de ecossistemas
aquáticos. Oecol Bras.

[B19] Majer BJ, Grummt T, Uhl M, Knasmuller S (2005). Use of plant bioassays for the detection of genotoxins in the aquatic
environment. Acta Hydrochim Hydrobiol.

[B20] Mielli AC, Matta MEM, Nersesyanc A, Saldiva PHN, Umbuzeiro GA (2009). Evaluation of the genotoxicity of treated urban sludge in the
*Tradescantia* micronucleus assay. Mutat Res.

[B21] Minas Gerais (2008). Deliberação Normativa n. 01 maio de 2008.

[B22] Misík M, Micieta K, Solenska M, Misikova K, Pisarcikova H, Knasmuller S (2007). In situ biomonitoring of the genotoxic effects of mixed industrial
emissions using the *Tradescantia* micronucleus and pollen abortion
tests with wild life plants: Demonstration of the efficacy of emission controls in
an eastern European city. Environ Pollut.

[B23] Monarca S, Feretti D, Zanardini A, Falistocco E, Nardi G (1999). Monitoring of mutagens in urban air samples. Mutat Res.

[B24] Nunes EA, Lemos CT, Gavronski L, Moreira TN, Oliveira NCD, Silva J (2011). Genotoxic assessment on river water using different biological
systems. Chemosphere.

[B25] Obidoska G, Jasinska D (2008). Phytotoxicity and potential genotoxicity of Radiowo municipal
leachate. Land Reclamation.

[B26] Pereira BB, Campos EO, Morelli S (2013). In situ biomonitoring of the genotoxic effects of vehicular pollution
in Uberlândia, Brazil, using *Tradescantia* micronucleus
assay. Ecotoxicol Environ Safe.

[B27] Sanches SM, Silva CHTP, Campos SX, Vieira EM (2003). Pesticidas e seus respectivos riscos associados à contaminação da
água. Ecotoxicol Meio Ambiente.

